# Prognostic model for predicting recurrence in hepatocellular carcinoma patients with high systemic immune-inflammation index based on machine learning in a multicenter study

**DOI:** 10.3389/fimmu.2024.1459740

**Published:** 2024-09-09

**Authors:** Ningning Lu, Shugui Sheng, Yiqi Xiong, Chuanren Zhao, Wenying Qiao, Xiaoyan Ding, Jinglong Chen, Yonghong Zhang

**Affiliations:** ^1^ Interventional Therapy Center for Oncology, Beijing You’an Hospital, Capital Medical University, Beijing, China; ^2^ Beijing Research Center for Respiratory Infectious Diseases, Beijing You’an Hospital, Capital Medical University, Beijing, China; ^3^ Beijing Key Laboratory of Emerging Infectious Diseases, Institute of Infectious Diseases, Beijing Ditan Hospital, Capital Medical University, Beijing, China; ^4^ Department of Cancer Center, Beijing Ditan Hospital, Capital Medical University, Beijing, China

**Keywords:** hepatocellular carcinoma, ablation therapy, recurrence-free survival, systemic immune-inflammation index, nomogram

## Abstract

**Introduction:**

This study aims to use machine learning to conduct in-depth analysis of key factors affecting the recurrence of HCC patients with high preoperative systemic immune-inflammation index (SII) levels after receiving ablation treatment, and based on this, construct a nomogram model for predicting recurrence-free survival (RFS) of patients.

**Methods:**

This study included clinical data of 505 HCC patients who underwent ablation therapy at Beijing You’an Hospital from January 2014 to January 2020, and accepted 65 HCC patients with high SII levels from Beijing Ditan Hospital as an external validation cohort. 505 patients from Beijing You’an Hospital were divided into low SII and high SII groups based on the optimal cutoff value of SII scores. The high SII group was further randomly divided into training and validation cohorts in a 7:3 ratio. eXtreme Gradient Boosting (XGBoost), random survival forest (RSF), and multivariate Cox regression analysis, were used to explore the factors affecting the post-ablation RFS of HCC patients. Based on the identified key factors, a nomogram model were developed to predict RFS in HCC patients, and their performance were evaluated using the concordance index (C index), receiver operating characteristic curve (ROC), calibration curve, and decision curve analysis (DCA). The optimal cutoff value for nomogram scores was used to divide patients into low- and high-risk groups, and the effectiveness of the model in risk stratification was evaluated using Kaplan-Meier (KM) survival curves.

**Results:**

This study confirmed that age, BCLC stage, tumor number, and GGT level were independent risk factors affecting RFS in HCC patients. Based on the selected risk factors, an RFS nomogram was successfully constructed. The C-index, ROC curve, calibration curve, and DCA curve each demonstrated the discrimination, accuracy, and decision-making utility of the nomogram, indicating that it has good predictive performance. KM curve revealed the nomogram could significantly differentiate patient populations with different recurrence risk.

**Conclusion:**

We developed a reliable nomogram that can accurately predict the 1-, 3-, and 5-year RFS for HCC patients with high SII levels following ablation therapy.

## Introduction

Primary liver cancer, predominantly hepatocellular carcinoma (HCC), is a lethal tumor of the digestive system that severely threatens global health. In 2020, statistics revealed 905,677 new cases worldwide and 830,180 deaths (1), with Asia accounting for 72% of these new cases and China alone exceeding half of the global total (2, 3). HCC constitutes 90% of primary liver cancers, and in early-stage treatments, ablation procedures have become a pivotal method, significantly controlling tumor progression (4). However, the high rate of postoperative recurrence undermines patients’ quality of life and prognosis, posing ongoing challenges to long-term survival (5–7). Consequently, predicting postoperative recurrence in HCC patients is crucial for personalized therapies and enhancing prognosis.

In recent years, the role of immune inflammation in the development, progression, and treatment of tumors has garnered significant attention (8–10). The interaction between the immune system and tumors is a complex and dynamic process, with the immune status of the organism playing a pivotal role in tumor proliferation, metastasis, and response to therapeutic interventions. Therefore, assessing the preoperative immune-inflammatory status of patients can provide new insights and approaches for clinical prediction. Against this backdrop, the systemic immune-inflammation index (SII), as a comprehensive indicator reflecting the overall immune-inflammatory state, has received extensive interest in cancer research (11–16). The SII can be readily obtained through routine blood tests, incorporating counts of lymphocytes, neutrophils, and platelets in the peripheral blood. SII = platelet count × neutrophil count/lymphocyte count (17). Not only is it simple to perform and cost-effective, but it also demonstrates good reproducibility. Clinically, SII has been confirmed to be closely associated with the prognosis of various cancers, with high levels of SII indicating an unfavorable outcome for the disease.

However, to date, there have been no prognostic model studies focusing on the recurrence in the specific population (HCC patients with high preoperative SII level) following ablation therapy. This group may exhibit more complex clinical characteristics and biological features, with their postoperative treatment responses and prognosis potentially differing from those of general HCC patients. Therefore, our research aims to identify the key factors influencing recurrence of HCC patients with high preoperative SII levels after the ablation treatment and establish the corresponding prognostic model.

## Materials and methods

### Study population

**Figure 1:**
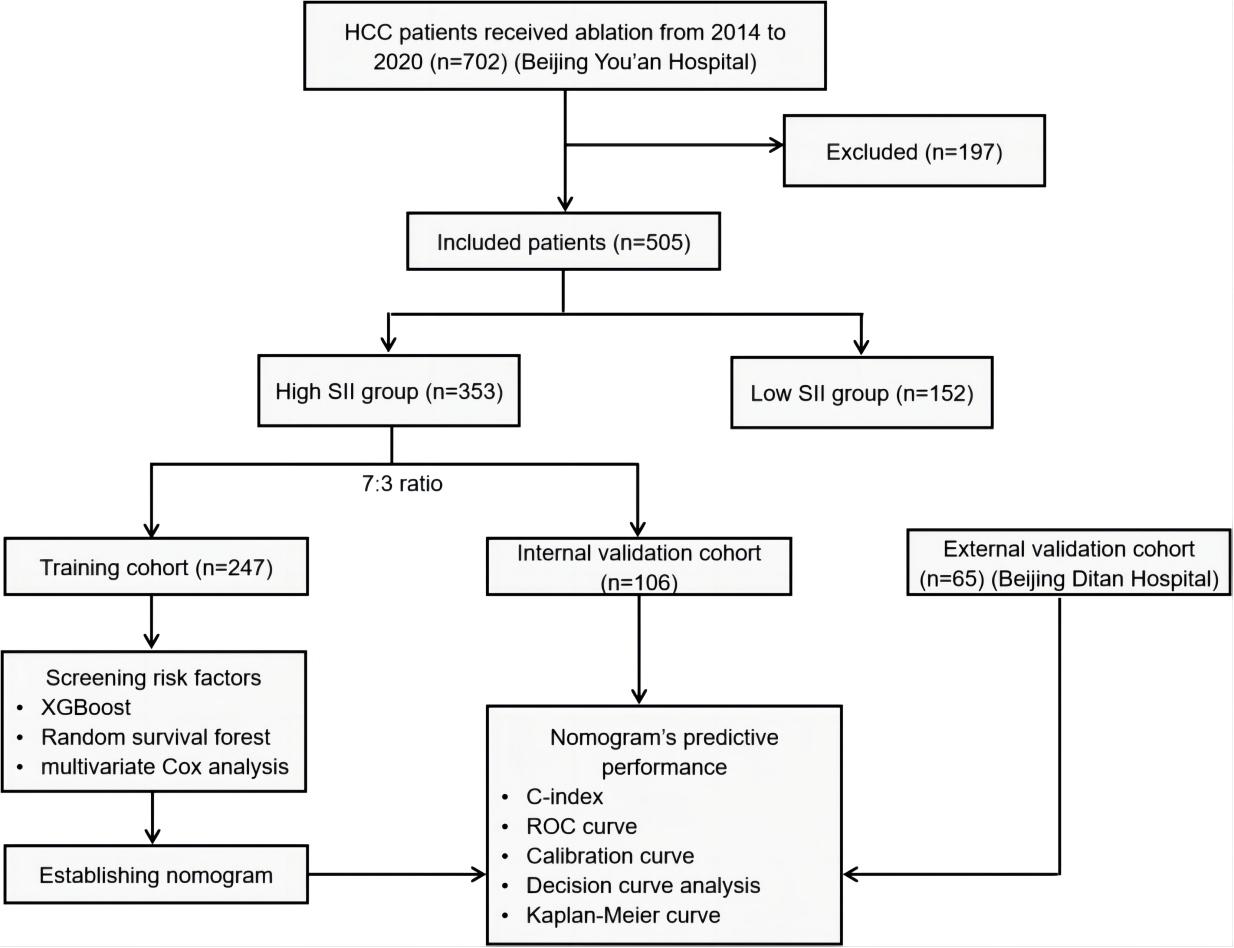
Patient enrollment and analysis workflow. HCC, hepatocellular carcinoma; SII, systemic immune-inflammation index; XGBoost, eXtreme gradient boosting; ROC, Receiver Operating Characteristic Curve.

This study adhered to clear inclusion and exclusion criteria, screening HCC patients who underwent ablation therapy at Beijing You’an Hospital affiliated to Capital Medical University, between January 2014 and January 2020. Ultimately, 505 patients were included. Additionally, a cohort comprising 65 HCC patients with high SII levels who underwent ablation therapy at Beijing Ditan Hospital affiliated to Capital Medical University, was incorporated for external validation. The patient enrollment and analysis workflow of this study is illustrated in **Figure 1**.

Inclusion Criteria (1): Patients diagnosed with HCC based on clinical symptoms, signs, and confirmed through ancillary tests such as laboratory examinations and imaging studies; (2) Patients who underwent ablation therapy; (3) Patients who had not received any other form of HCC-related treatment prior to the therapy; (4) Patients without evident advanced hepatic insufficiency before treatment; (5) Patients with complete medical records and follow-up data. Exclusion Criteria: (1) Patients diagnosed with non-primary HCC; (2) Patients who received other anti-HCC treatments prior to ablation therapy; (3) Patients with significant dysfunction in major organs such as heart, lung, or kidney; (4) Patients with severe immunodeficiency or autoimmune diseases; (5) Patients with incomplete follow-up data or for whom data cannot be obtained.

### Data collection

In this study, we collected baseline characteristic data of patients prior to their ablation treatment, encompassing multiple dimensions including: (1) Personal information: age and gender; (2) Medical history: hypertension, diabetes, smoking history, family history, and antiviral treatment history; (3) Imaging and pathological features: cirrhosis, Child-Pugh class, Barcelona Clinic Liver Cancer (BCLC) stage, tumor number, and tumor size; (4) Laboratory test indicators: white blood cells (WBC), monocytes, red blood cells (RBC), eosinophils, basophils, hemoglobin (Hb), alanine aminotransferase (ALT), aspartate aminotransferase (AST), total bilirubin (TBIL), direct bilirubin (DBIL), total protein (TP), albumin, globulin, gamma-glutamyl transpeptidase (GGT), alkaline phosphatase (ALP), prealbumin, bile acid, blood urea nitrogen (BUN), creatinine, uric acid, glucose, cholesterol, potassium ions, sodium ions, chloride ions, prothrombin time (PT), international normalized ratio (INR), activated partial thromboplastin time (APTT), fibrinogen, thrombin time (TT), and alpha-fetoprotein (AFP). By collecting and analyzing these diverse data, we aim to identify potential factors and characteristics associated with the prognosis of HCC patients with high SII levels.

### Ablation procedure and follow-up

The majority of patients in this study were primarily treated with radiofrequency ablation (RFA), while a small number received microwave ablation (MWA) therapy. The core mechanism of RFA involves the use of ultrasound or CT guidance to precisely insert a radiofrequency electrode into tumor tissue. The frequency waves emitted by the radiofrequency electrode stimulate polar molecules and ions in the tumor tissue, causing them to move and oscillate at high speeds in sync with the frequency of the radiofrequency current, thereby generating frictional heat. This heat is conducted to adjacent tissues, resulting in an increase in temperature within the tumor tissue. As water inside and outside the cells evaporates, dries up, and contracts, the tumor tissue undergoes aseptic necrosis, achieving the therapeutic objective. The principle of MWA is to accurately locate cancer lesions through CT or ultrasound guidance and directly target the core of the tumor with a specially designed microwave ablation needle. The “miniature microwave oven” at the tip of the microwave ablation needle releases a microwave electromagnetic field, causing water molecules, protein molecules, and other components within the tumor tissue to vibrate rapidly and rub against each other, generating high temperatures swiftly. This leads to irreversible coagulation necrosis and dehydration of HCC cells, achieving the goal of thermal ablation.

After the patient underwent ablation treatment, the hospital recommended regular follow-up visits. The initial follow-up was usually scheduled about one month after the treatment to assess its efficacy. Subsequent follow-up plans suggested quarterly visits within the first year of treatment, which then transitioned to biannual visits until the patient experienced a recurrence or was lost to follow-up. RFS is defined as the duration from the date of ablation to the date of recurrence or the last follow-up date. The last follow-up date in this research is December 31, 2023.

### Statistical analysis

In this study, for continuous variables, we presented the data using means ± standard deviations; for categorical variables, we clearly displayed the proportion of each category through frequencies and percentages. We employed Student’s t-test to analyze between-group differences in continuous variables and Chi-square test to assess between-group differences in categorical variables. P value < 0.05 indicated statistical significance. Statistical analyses were conducted using R version 4.3.2. Initially, we calculated the SII values for 505 patients from Beijing You’an Hospital and categorized them into low SII (152 patients) and high SII (353 patients) groups based on an optimal cutoff value of SII = 197.84. Subsequently, patients in the high SII group were randomly divided into training and internal validation cohorts at a 7:3 ratio. To explore key factors influencing RFS after ablation therapy in HCC patients with high SII, we utilized statistical methods including XGBoost, RSF, and multivariate Cox regression. XGBoost and RSF algorithms excel in handling numerous feature variables and have strong resistance to overfitting, while multivariate Cox regression provides more accurate survival predictions by considering interactions among multiple predictive factors. Based on the identified critical factors, we developed a nomogram model for predicting RFS after ablation therapy. To evaluate the model’s performance, we adopted various statistical measures, including the concordance index (C-index), receiver operating characteristic curve (ROC), calibration curves, and decision curve analysis (DCA). The model’s performance was further validated using the external validation cohort of 65 high SII HCC patients from Beijing Ditan Hospital. Using the optimal cutoff value for nomogram scores, we classified patients into low-risk and high-risk groups. By comparing the Kaplan-Meier survival curves of these two groups, we assessed the nomogram’s effectiveness in distinguishing patients with different recurrence risk levels.

## Results

### Comparison of prognosis between preoperative low SII and high SII HCC patients receiving ablation treatment

**Figure 2:**
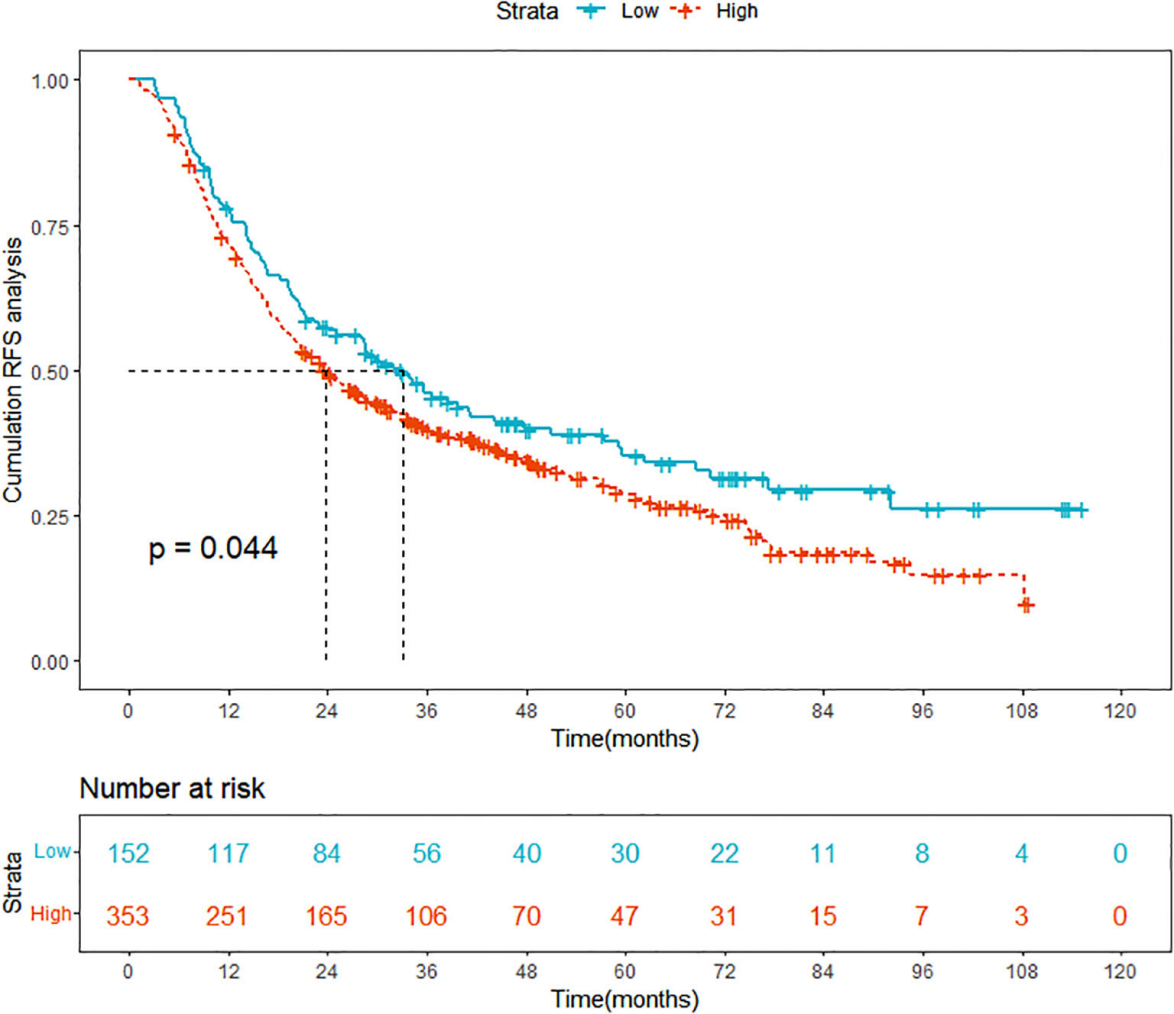
Comparison of Kaplan-Meier curves for prognosis between high SII and low SII HCC groups. SII, systemic immune-inflammation index; HCC, hepatocellular carcinoma; RFS, recurrence-free survival.

In this retrospective study, we finally enrolled 505 HCC patients who underwent ablation therapy from Beijing You’an Hospital and 65 high SII level HCC patients who underwent ablation therapy from Beijing Ditan Hospital and conducted a detailed collation and analysis of their clinical data. The 505 patients were divided into two groups based on the optimal cutoff value of the SII score, which was determined to be 197.8: the low SII group comprised 152 patients, while the high SII group included 353 patients. The KM curve demonstrated that the RFS was significantly lower in the high SII group compared to the low SII group, indicating a notably higher recurrence rate for patients in the high SII group (**Figure 2**). Therefore, there is an urgent clinical need to develop tailored predictive models for patients with high SII, to better inform treatment strategies and practices.

### Baseline characteristics analysis of high SII HCC patients

The objective of this study is to develop a predictive model for RFS following ablation therapy in high preoperative SII HCC patients, based on machine learning. We randomly allocated the 353 high SII group patients from Beijing You’an Hospital into two cohorts with a 7:3 ratio: a training cohort (n=247) and an internal validation cohort (n=106). To ensure the credibility of the results, comparative analyses were conducted to meticulously compare the baseline characteristics between the training cohort, the internal validation cohort and the external validation cohort (**Table 1**). In terms of age distribution, the mean age of the training cohort is 56.98 years, that of the internal validation cohort is 56.06 years, and that of the external validation cohort is 58.09 years, and the P value of 0.395 suggests that there is no significant difference in age distribution between the three cohorts. As for gender distribution, in the training cohort, males constitute 72.1%, and females 27.9%. In contrast, the internal validation cohort comprises 75.5% males and 24.5% females, implying a slightly higher proportion of males in both cohorts compared to females. Consolidating the analytical results of other characteristics, it is evident that there are no significant differences between the three cohorts in terms of age, gender, medical history, disease staging, tumor characteristics, as well as laboratory examination indicators. These outcomes furnish us with a solid data foundation for subsequent research, facilitating a deeper analysis into the risk factors influencing RFS after ablation therapy for HCC patients with high preoperative SII level.

**Table 1:** Baseline characteristics analysis of training, internal validation and external validation cohorts.

Characteristic	Training cohort(N=247)	Internal validation cohort(N=106)	External validation cohort(N=65)	*P* value
Gender (male/female)	178 (72.1%)/69 (27.9%)	80 (75.5%)/26 (24.5%)	48 (73.8%)/17 (26.2%)	0.797
Hypertension (no/yes)	181 (73.3%)/66 (26.7%)	77 (72.6%)/29 (27.4%)	48 (73.8%)/17 (26.2%)	0.984
Diabetes (no/yes)	206 (83.4%)/41 (16.6%)	88 (83.0%)/18 (17.0%)	55 (84.6%)/10 (15.4%)	0.962
Antiviral history (no/yes)	93 (37.7%)/154 (62.3%)	47 (44.3%)/59 (55.7%)	22 (33.8%)/43 (66.2%)	0.336
Smoking history (no/yes)	169 (68.4%)/78 (31.6%)	75 (70.8%)/31 (29.2%)	50 (76.9%)/15 (23.1%)	0.408
Family history (no/yes)	136 (55.1%)/111 (44.9%)	55 (51.9%)/51 (48.1%)	40 (61.5%)/25 (38.5%)	0.466
Cirrhosis (no/yes)	33 (13.4%)/214 (86.6%)	18 (17.0%)/88 (83.0%)	8 (12.3%)/57 (87.7%)	0.604
Child-Pugh class (A/B)	210 (85.0%)/37 (15.0%)	91 (85.8%)/15 (14.2%)	58 (89.2%)/7 (10.8%)	0.686
BCLC stage (0/A)	86 (34.8%)/161 (65.2%)	39 (36.8%)/67 (63.2%)	24 (36.9%)/41 (63.1%)	0.914
Tumor number (single/multiple)	166 (67.2%)/81 (32.8%)	81 (76.4%)/25 (23.6%)	45 (69.2%)/20 (30.8%)	0.223
Tumor size (≤3cm/>3cm)	162 (65.6%)/85 (34.4%)	68 (64.2%)/38 (35.8%)	42 (64.6%)/23 (35.4%)	0.964
Age	56.98 ± 9.12	56.06 ± 10.84	58.09 ± 8.83	0.395
Eosinophil (10^9/L)	0.12 ± 0.12	0.13 ± 0.19	0.14 ± 0.15	0.431
Basophil (10^9/L)	0.01 ± 0.02	0.01 ± 0.01	0.02 ± 0.02	0.091
ALT (U/L)	31.94 ± 20.23	32.75 ± 19.25	28.82 ± 13.38	0.399
AST (U/L)	30.64 ± 14.80	30.02 ± 12.06	29.54 ± 14.22	0.828
TBIL (μmol/L)	17.91 ± 8.97	18.08 ± 8.98	16.92 ± 8.34	0.675
DBIL (μmol/L)	5.88 ± 4.22	5.80 ± 3.45	5.26 ± 3.05	0.521
Albumin (g/L)	37.86 ± 4.18	38.20 ± 4.36	37.80 ± 4.30	0.76
Globulin (g/L)	27.62 ± 4.72	27.54 ± 5.29	27.61 ± 3.90	0.99
GGT (U/L)	58.03 ± 45.69	63.62 ± 55.57	55.86 ± 36.68	0.495
ALP (U/L)	84.27 ± 31.43	82.81 ± 26.31	82.68 ± 25.42	0.874
Prealbumin (g/L)	141.95 ± 55.99	150.45 ± 62.92	129.34 ± 48.78	0.063
Creatinine (μmol/L)	69.47 ± 67.37	67.01 ± 18.41	66.04 ± 14.35	0.861
Uric acid (μmol/L)	275.40 ± 84.72	289.20 ± 84.80	278.98 ± 85.11	0.375
Glucose (mmol/L)	6.00 ± 2.14	5.92 ± 1.94	5.88 ± 2.36	0.899
Potassium (mmol/L)	3.98 ± 0.41	3.96 ± 0.36	3.91 ± 0.40	0.45
Sodium (mmol/L)	139.71 ± 2.77	139.60 ± 2.54	140.25 ± 2.65	0.27
Chlorine (mmol/L)	103.36 ± 3.18	103.41 ± 3.02	103.15 ± 2.95	0.862
PT (s)	12.42 ± 1.30	12.19 ± 1.24	12.47 ± 1.45	0.247
APTT (s)	32.77 ± 4.79	31.98 ± 3.69	33.06 ± 6.07	0.064
Fibrinogen (g/L)	3.02 ± 0.95	3.12 ± 1.01	3.08 ± 1.00	0.688
TT (s)	15.45 ± 2.11	15.13 ± 2.06	15.45 ± 2.10	0.402
AFP (ng/mL)	475.83 ± 2194.13	294.05 ± 858.96	163.48 ± 431.81	0.371

BCLC, Barcelona Clinic Liver Cancer; ALT, alanine aminotransferase; AST, aspartate aminotransferase; TBIL, total bilirubin; DBIL, direct bilirubin; GGT, gamma glutamyl transpeptidase; ALP, alkaline phosphatase; PT, prothrombin time; APTT, activated partial thromboplastin time; TT, thrombin time; AFP, alpha-fetoprotein.

### Identifying factors affecting RFS in high SII HCC patients

**Figure 3:**
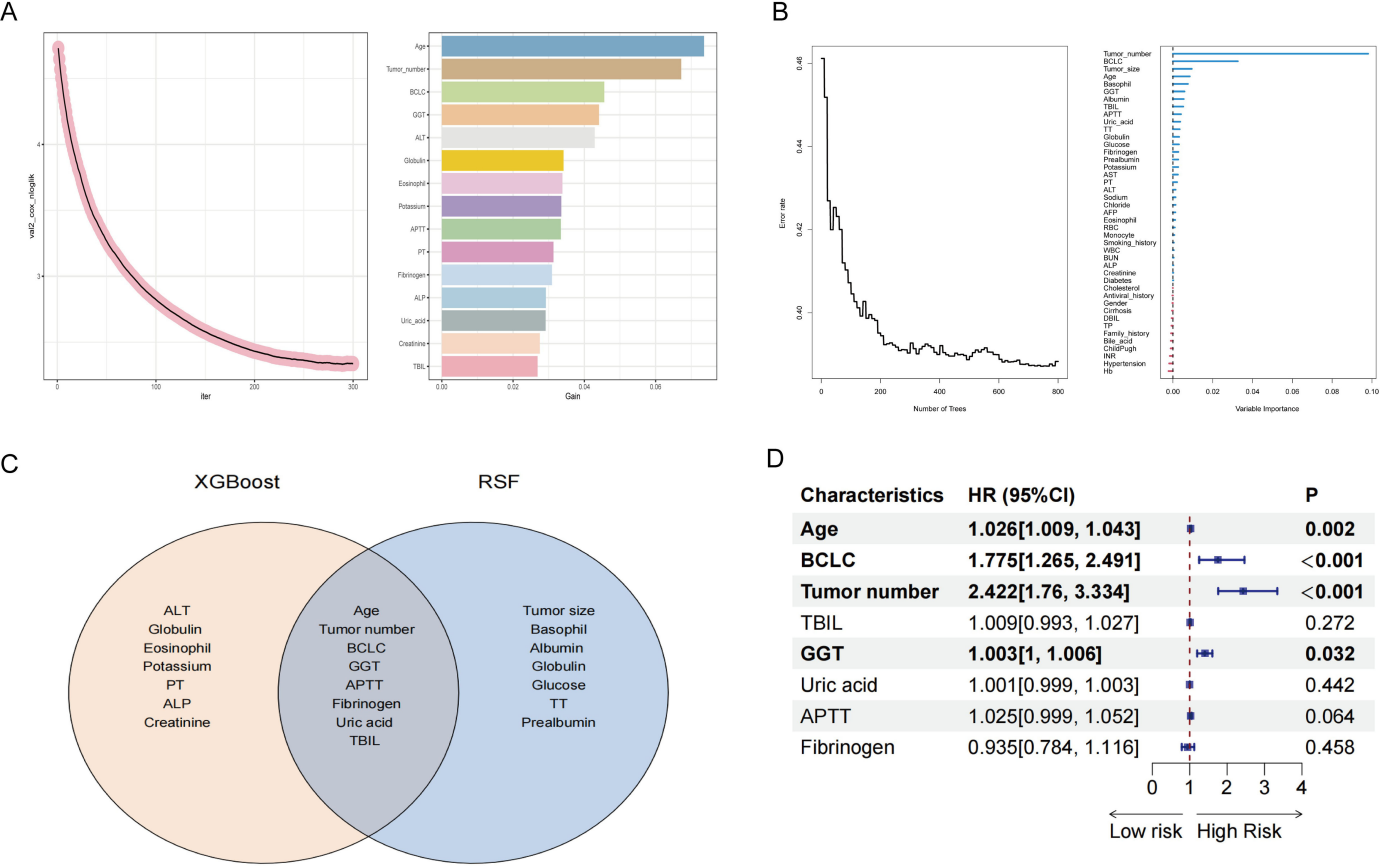
Screening for factors affecting post-ablation RFS in HCC patients with high SII. **(A)** Using XGBoost to screen factors. **(B)** Using random survival forest to screen factors. **(C)** Intersection of XGBoost and random survival forest screening results. **(D)** Multivariate Cox regression analysis. SII, systemic immune-inflammation index; HCC, hepatocellular carcinoma; RFS, recurrence-free survival; XGBoost, eXtreme gradient boosting; BCLC, Barcelona Clinic Liver Cancer; GGT, gamma glutamyl transpeptidase; TBIL, total bilirubin; APTT, activated partial thromboplastin time; TT, thrombin time; AST, aspartate aminotransferase; PT, prothrombin time; ALT, alanine aminotransferase; AFP, alpha-fetoprotein; RBC, red blood cell; WBC, white blood cell; BUN, blood urea nitrogen; ALP, alkaline phosphatase; DBIL, direct bilirubin; TP, total protein; INR, international normalized ratio; Hb, hemoglobin.

This section comprehensively uncovers potential risk factors influencing the RFS of high SII HCC patients following ablation therapy, by integrating machine learning techniques (XGBoost and random survival forest) with classical statistical analyses (multivariate Cox regression). We initially employed the XGBoost algorithm for preliminary variable selection. Renowned for its efficiency in handling complex datasets, the XGBoost algorithm is particularly adept at capturing nonlinear relationships and interactions within the data. Using this algorithm, we have successfully identified a set of variables that have significant impacts on RFS. **Figure 3A** visually illustrates the top 15 key variables selected by the XGBoost algorithm, ranked by their importance. These include age, tumor number, BCLC stage, GGT, ALT, globulin, eosinophils, potassium ions, APTT, PT, fibrinogen, ALP, uric acid, creatinine, and TBIL.

In addition to employing the XGBoost algorithm, we have also introduced the random survival forest (RSF) algorithm for analyzing variables impacting RFS. Compared to XGBoost, the RSF algorithm holds distinct advantages when dealing with survival data. The RSF method enhances model diversity by constructing multiple survival trees through random selection of both samples and variables during training. In **Figure 3B**, we present the results of the RSF analysis, with the top 15 important variables being: tumor number, BCLC stage, tumor size, age, basophils, GGT, albumin, TBIL, APTT, uric acid, TT, globulin, glucose, fibrinogen, and prealbumin.

Subsequently, we performed an intersection analysis on the top 15 important variables screened from both XGBoost and RSF methodologies. This step aimed to identify common variables that exhibited significant importance in both approaches, thereby enhancing our confidence in these variables and laying a more robust foundation for subsequent model construction. **Figure 3C** clearly illustrates these commonly important variables, comprising age, tumor number, BCLC stage, GGT, APTT, fibrinogen, uric acid, and TBIL.

Finally, we conducted a more rigorous screening process using multivariate Cox regression analysis. Multivariate Cox regression analysis is a commonly employed statistical method used to evaluate the impact of multiple variables on survival time. By controlling for potential confounding factors, we can precisely measure the weight of influence of each variable, thereby identifying independent risk factors among them. As shown in **Figure 3D**, through multivariate Cox analysis, we confirmed that age, BCLC stage, tumor number, and GGT level are independent risk factors affecting RFS. This finding not only enhances our understanding of the factors influencing RFS but also provides crucial groundwork for constructing predictive nomogram for RFS in the following section.

### Construction of RFS prognostic nomogram in high SII HCC patients

**Figure 4:**
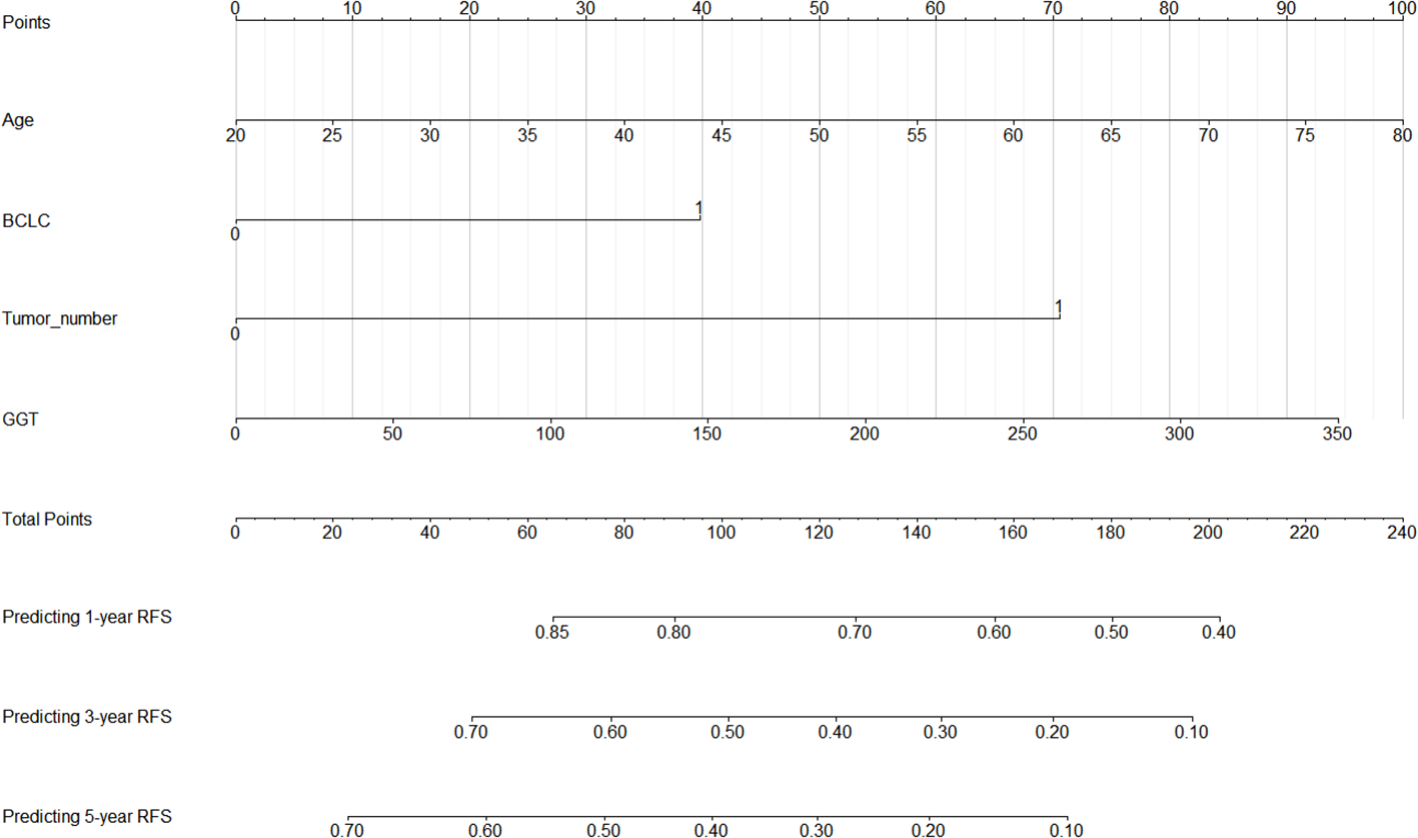
Nomogram for 1-, 3-, and 5-year post-ablation RFS prediction in HCC patients with high SII. SII, systemic immune-inflammation index; HCC, hepatocellular carcinoma; RFS, recurrence-free survival; BCLC, Barcelona Clinic Liver Cancer; GGT, gamma glutamyl transpeptidase.

Based on the analysis results from the previous section, we have identified age, BCLC stage, tumor number, and GGT as independent risk factors for RFS. To visually illustrate the specific impact of these factors on RFS, we have constructed a nomogram, as detailed in **Figure 4**. First, for each variable, an appropriate point is assigned based on its importance and the extent of its impact. Then, these points are aggregated to derive the total points. Following this, the total points are matched against a pre-established outcome scale, which yields the probability of a certain clinical event occurring, such as the probability of HCC recurrence in this study.

**Figure 5:**
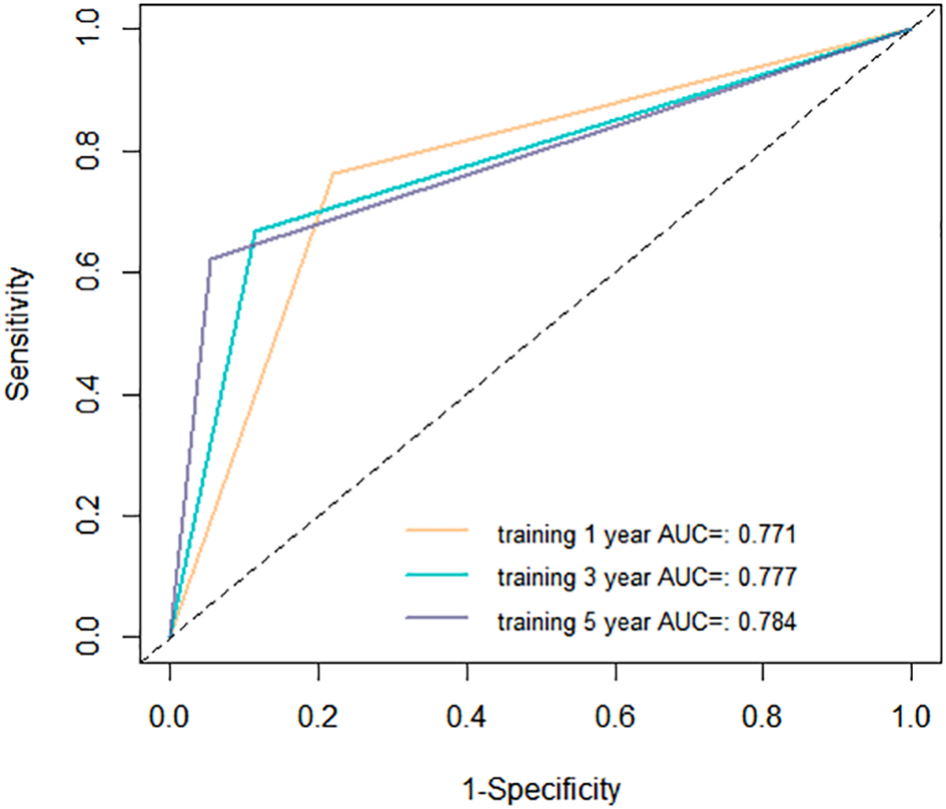
Receiver operating characteristic (ROC) curves of the nomogram in the training cohort. AUC, area under the curve.

The aim of a nomogram prediction model is to predict outcomes as accurately as possible; hence, a nomogram is evaluated based on three crucial metrics: discrimination, calibration, and clinical utility. Discrimination is an index to evaluate a nomogram’s ability to distinguish between patients who have experienced positive events and those who have not. This evaluative metric is measured using two primary measures: the C-index and the ROC curve. The nomogram model we established achieved a C-index of 0.731 (95% CI: 0.688-0.774) in the training cohort, indicating that our model can effectively distinguish between individuals who experience recurrence and those who do not. Furthermore, we depicted ROC curves for 1-year, 3-year, and 5-year RFS in the training cohort, as shown in **Figure 5**. The findings revealed that the AUC values for predicting 1-year, 3-year, and 5-year RFS were 0.771, 0.777, and 0.784, respectively, demonstrating that our model performs well in predicting RFS.

**Figure 6:**
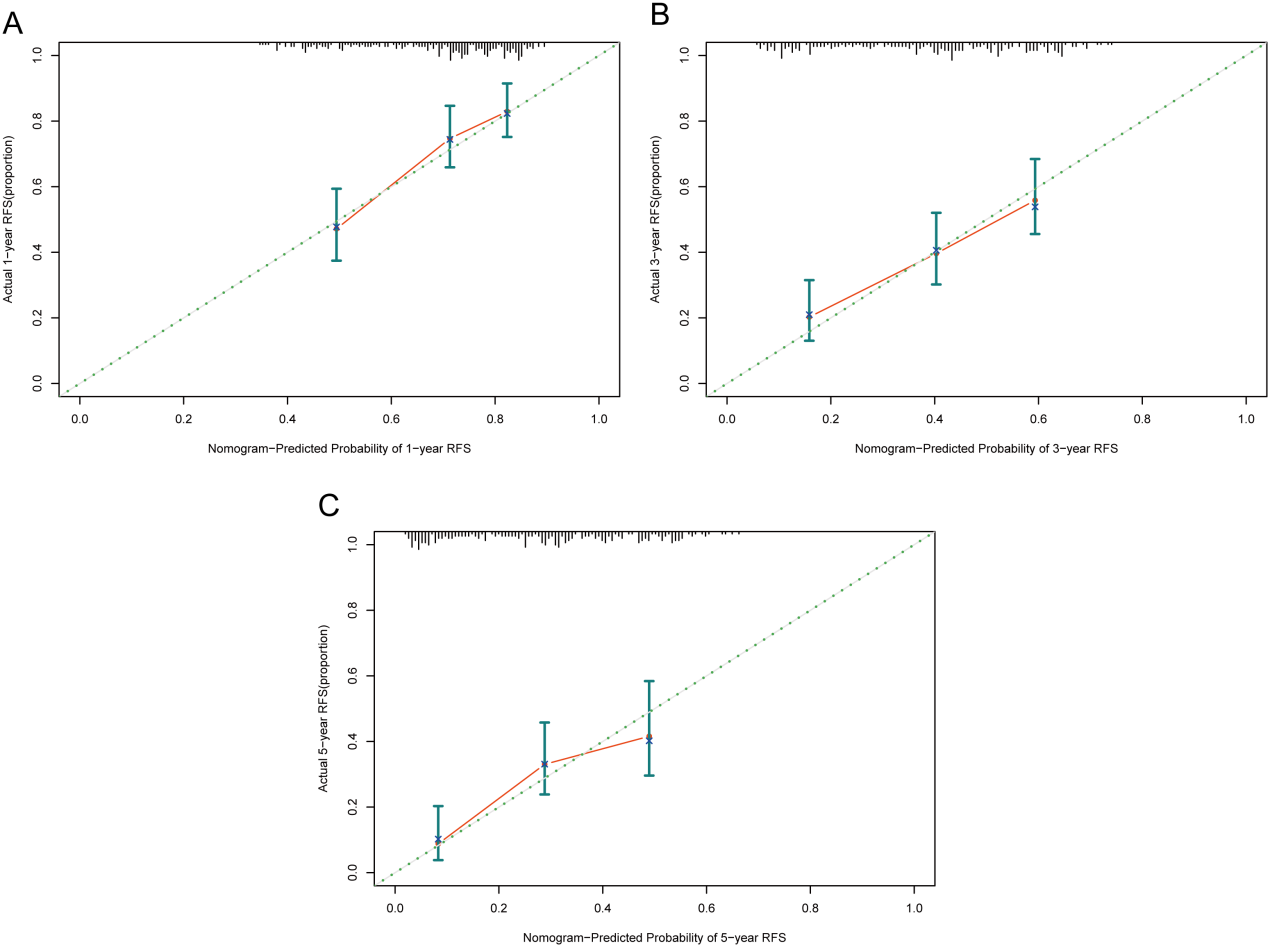
Calibration curves of the nomogram in the training cohort. **(A)** 1-year calibration curve. **(B)** 3-year calibration curve. **(C)** 5-year calibration curve. RFS, recurrence-free survival.

Calibration assesses the degree of deviation between a model’s predicted outcomes and the actual outcomes. It can be evaluated through a calibration curve, which depicts the relationship between the probabilities predicted by the model and the actual probabilities of events occurring. As shown in **Figure 6**, the proximity between the solid line (model-predicted probabilities) and the dashed line (actual occurrence probabilities) in our calibration curve indicates a strong agreement between the predicted probabilities of 1-year, 3-year, and 5-year RFS by our model and the probabilities actually observed for the patients.

**Figure 7:**
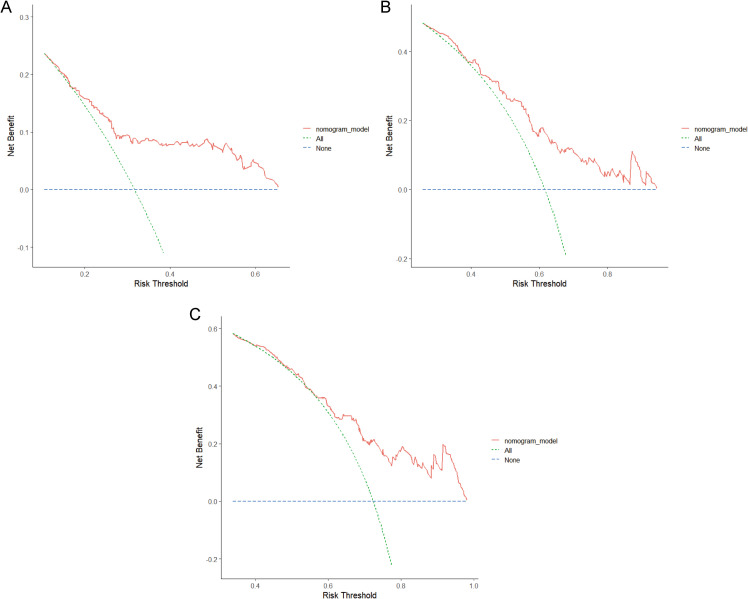
Decision curve analysis (DCA) of the nomogram in the training cohort. **(A)** 1-year DCA curve. **(B)** 3-year DCA curve. **(C)** 5-year DCA curve. RFS, recurrence-free survival.

Clinical utility is typically analyzed using the DCA cure, which evaluates the clinical effectiveness of a predictive model based on varying threshold probabilities. In the DCA graph, the x-axis represents the risk threshold, while the y-axis denotes the net benefit. The DCA graph illustrates two extreme scenarios. In one scenario, the curve is flat, indicating that if no patients receive treatment, the net benefit rate is zero. The other scenario is represented by a sloped curve, which signifies that assuming all samples test positive, and all individuals undergo intervention, the net benefit rate is a negatively sloped line. When the DCA curve of a predictive model closely aligns with these two extremes, it suggests that the model’s utility in actual clinical decision-making is limited. Conversely, if the DCA curve significantly surpasses the extreme curves, this indicates a higher potential for clinical application of the predictive model. **Figure 7** illustrates the net benefit of the nomogram over a range of threshold probabilities for 1-year, 3-year, and 5-year RFS, demonstrating significant clinical benefit.

### Internal validation of RFS prognostic nomogram

We performed internal validation of the nomogram model using the internal validation cohort from Beijing You’an Hospital, confirming its ability to provide accurate predictions and demonstrating its effectiveness for clinical application. The AUC values for 1-year, 3-year, and 5-year RFS in the internal validation cohort were 0.733, 0.806, and 0.780, respectively, which further substantiates the accuracy and reliability of the nomogram in predicting patient recurrence (**Supplementary Figure S1**). **Supplementary Figure S2** illustrates that in the internal validation cohort, the calibration curves for 1-year, 3-year, and 5-year predictions closely align with the ideal 45°diagonal, indicating that the predicted probabilities from our nomogram are highly consistent with the actual observed outcomes. **Supplementary Figure S3** demonstrates that the DCA curves for 1-year, 3-year, and 5-year RFS exhibit a high net benefit across a broad range of probability thresholds, further emphasizing the clinical utility of our model.

### External validation of RFS prognostic nomogram

In this section, we present the external validation process for the RFS prognostic nomogram using the 65 high SII HCC patients data from Beijing Ditan Hospital. This validation step is crucial to ensure that the nomogram can be reliably applied to new patients in another institution. The 1-year, 3-year, and 5-year AUC values for the nomogram in the external dataset were 0.703, 0.716, and 0.732, respectively (**Supplementary Figure S4**). The calibration plot demonstrated good agreement between the predicted and observed RFS probabilities, with the predicted curve closely overlapping the 45-degree line of perfect prediction (**Supplementary Figure S5**). The DCA curve demonstrated that the nomogram provided a higher net benefit compared to either treating all patients or treating none, across a wide range of threshold probabilities (**Supplementary Figure S6**). The high AUC value, favorable calibration, and positive DCA results collectively suggest that the RFS prognostic nomogram performs well in the external validation setting.

### Risk stratification capability of RFS prognostic nomogram

**Figure 8:**
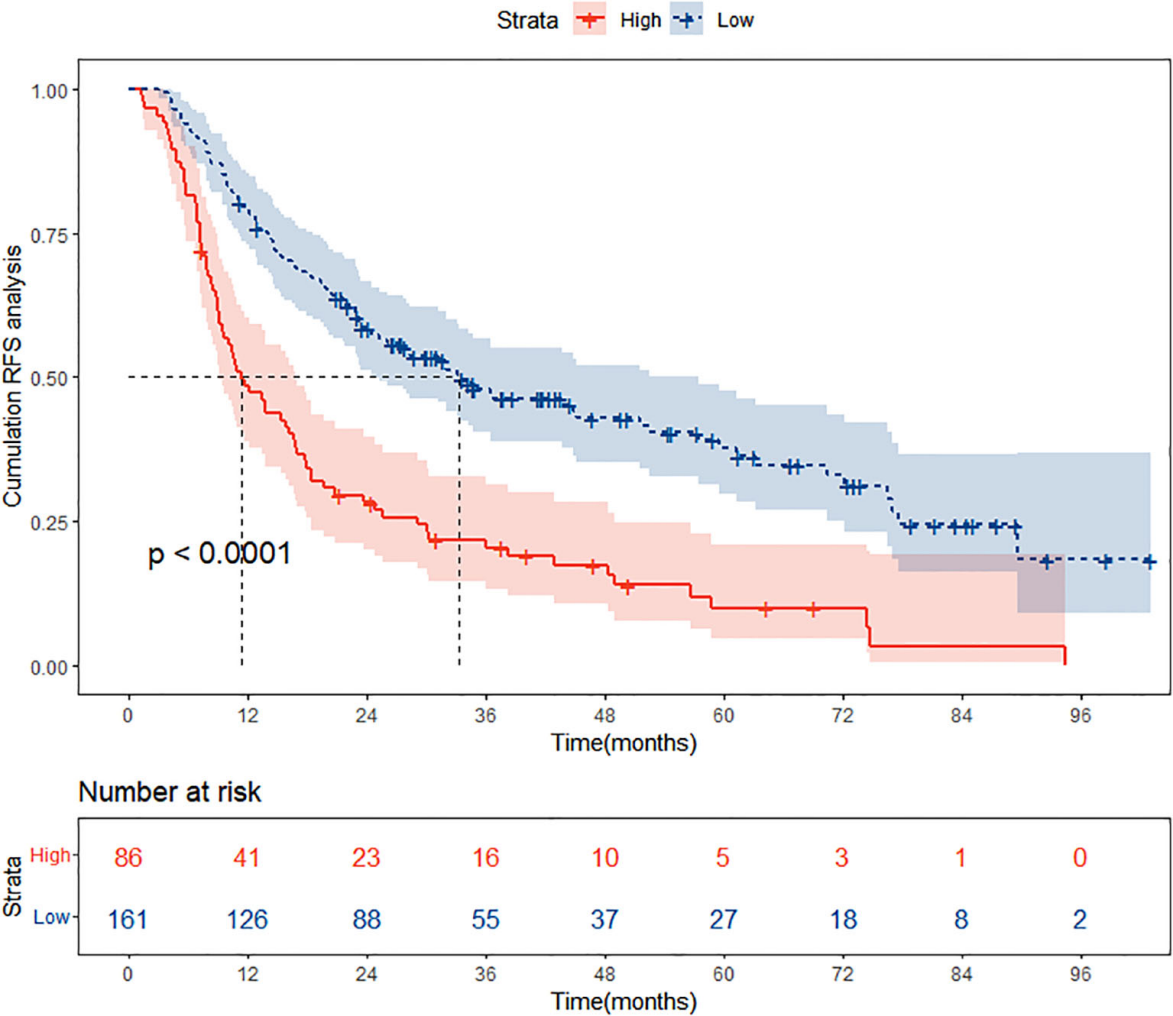
Kaplan Meier curve analysis of different risk groups stratified by nomogram scores in the training cohort. RFS, recurrence-free survival.

In the training cohort, based on the optimal cut-off value derived from the nomogram points, patients were stratified into two distinct risk groups: low-risk and high-risk. This segmentation aims to evaluate the nomogram’s efficacy in accurately discriminating between populations with different recurrence risk profiles. In this section, Kaplan-Meier curves were employed to compare the RFS between the low-risk and high-risk groups. As depicted in **Figure 8**, a significant difference in recurrence risk is evident between the low-risk and high-risk groups within the training cohort (P<0.05), with patients in the low-risk group exhibiting significantly longer RFS compared to those in the high-risk group. This suggests that low-risk group patients have a more favorable prognosis, encountering a lower risk of recurrence relative to high-risk group patients. Conversely, the more rapid decline in RFS observed in the high-risk group indicates a higher propensity for recurrence among these patients.

## Discussion

In the clinical practice of HCC, accurately predicting patient prognosis is crucial for devising effective treatment strategies. However, traditional prognostic models often fail to adequately consider the distinctiveness of patients with high SII levels, whose immune-inflammatory status is closely linked to treatment response and outcomes. This study is dedicated to addressing this gap by introducing innovative analytical approaches and focusing on the specific patient population, aiming to provide more precise and reliable RFS prediction tools for HCC patients with high SII levels. By leveraging machine learning algorithms, we were able to capture complex non-linear relationships between variables, thereby improving the accuracy of RFS predictions in HCC patients with high SII levels.

Previous studies have shown that the prognosis of HCC is influenced by a complex interplay of factors that collectively impact the disease course and treatment outcomes in patients. These factors encompass host-related elements, clinical-pathological aspects, laboratory indicators, and molecular biological considerations, among others. Regarding host-related factors, age, gender, and underlying health status can significantly affect its prognosis (18–21). For instance, gender disparities can lead to differing prognostic outcomes, possibly linked to hormonal levels and lifestyle factors. Shen et al. investigated diabetes’ impact on HCC patients’ post-hepatectomy prognosis, focusing on late recurrences beyond two years to precisely gauge long-term survival effects (22). Applying Kaplan-Meier analysis, they observed significantly reduced 3- and 5-year overall survival (OS) rates in diabetic versus non-diabetic patients. Cox regression affirmed diabetes as an independent risk factor for OS and RFS. Kobayashi et al. explored the impact of reduced muscle mass on the prognosis of HCC patients receiving transarterial chemoembolization (TACE) (23). Their study findings revealed that patients with a significant decrease in skeletal muscle mass had a notably lower OS compared to those without a marked reduction in muscle mass. In another study conducted by Dou et al., they aimed to identify the body mass index (BMI) ranges that were associated with prolonged survival in patients with HCC who underwent MWA (24). Their findings revealed that, for HCC patients undergoing MWA treatment, the optimal BMI range for survival was between 21.5 and 23.1 kg/m².

Beyond host-related factors, clinical-pathological elements are also crucial in shaping HCC prognosis. Tumor size, number, and presence of vascular invasion are pivotal indicators in assessing HCC outcomes. These clinical-pathological features not only reflect the malignancy and biological behavior of the tumor but also inform therapeutic planning. Laboratory indicators also hold considerable value in evaluating HCC prognosis. Inflammatory-immune markers (25, 26), liver function tests (27, 28), and tumor biomarkers (29–31) provide substantial support for prognosis assessment. Changes in these laboratory indicators not only reflect the patient’s physiological and pathological state but also indicate tumor progression and treatment response. Abnormal liver function tests often signal liver damage and disease progression, whereas alterations in inflammatory-immune markers may relate to tumor immune evasion and recurrence risk. Molecular biological factors, as an emerging area in HCC prognosis evaluation, have garnered significant attention in recent years. Gene expression levels (32, 33), non-coding RNAs (34, 35), and circulating tumor cells (36–39) offer new insights and approaches to HCC prognosis. These molecular biological factors not only elucidate the molecular mechanisms underlying HCC development but also provide foundations for novel therapeutic strategies and drug development.

Our research highlighted the critical roles of age, BCLC stage, tumor number, and GGT levels in determining the prognosis of HCC patients with high SII. Age, as a non-modifiable factor, influences the overall health status and comorbidity profile, impacting treatment tolerance and outcomes. The BCLC staging system, widely recognized for its prognostic value, provides a comprehensive assessment of tumor burden and liver function, directly affecting the choice of therapy and survival rates. The tumor number is a direct indicator of disease extent, with higher counts generally correlating with poorer prognosis. Lastly, GGT reflects the underlying liver disease severity and high GGT level correlates with more severe liver dysfunction and worse prognosis.

While our model represents a significant advancement, it is not without limitations. Firstly, the model’s performance and generalizability may be constrained by the size and diversity of the dataset used for training and validation. Larger and more diverse datasets can help improve the model’s predictive accuracy. For instance, a more extensive dataset would include patients from various geographic regions, and ethnic backgrounds. This would allow the model to better account for the potential influence of these demographic factors on health outcomes. Secondly, the clinical features considered in the model may not capture all the relevant variables that could affect HCC outcomes. For example, socioeconomic factors, lifestyle habits, and other comorbid conditions could play a role in HCC progression but were not included due to data limitations. These omitted variables might introduce unmeasured confounding, affecting the accuracy of the predictions. To address this limitation, future studies should consider incorporating a wider range of clinical, environmental, and social factors that could potentially influence HCC outcomes. Lastly, integrating molecular and genetic markers into prognostic models might offer even greater precision in predicting HCC outcomes. For example, genetic mutations such as those in the TERT promoter or telomerase activity levels, and molecular signatures like microRNA expression profiles, have been linked to HCC prognosis. Incorporating such data could enable personalized medicine approaches, tailoring treatments to the unique molecular characteristics of each patient’s tumor.

## Conclusion

We developed a reliable nomogram that can accurately predicts the 1-, 3-, and 5-year RFS for HCC patients with high SII levels following ablation therapy.

## Data Availability

The original contributions presented in the study are included in the article/**Supplementary Material**. Further inquiries can be directed to the corresponding authors.

## Supplementary material

Supplementary Figure 1 Receiver operating characteristic (ROC) curves of the nomogram in the internal validation cohort. AUC, area under the curve.

Supplementary Figure 2 Calibration curves of the nomogram in the internal validation cohort. **(A)** 1-year calibration curve. **(B)** 3-year calibration curve. **(C)** 5-year calibration curve. RFS, recurrence-free survival.

Supplementary Figure 3 Decision curve analysis (DCA) of the nomogram in the internal validation cohort. **(A)** 1-year DCA curve. **(B)** 3-year DCA curve. **(C)** 5-year DCA curve. RFS, recurrence-free survival.

Supplementary Figure 4 Receiver operating characteristic (ROC) curves of the nomogram in the external validation cohort. AUC, area under the curve.

Supplementary Figure 5 Calibration curves of the nomogram in the external validation cohort. **(A)** 1-year calibration curve. **(B)** 3-year calibration curve. **(C)** 5-year calibration curve. RFS, recurrence-free survival.

Supplementary Figure 6 Decision curve analysis (DCA) of the nomogram in the external validation cohort. **(A)** 1-year DCA curve. **(B)** 3-year DCA curve. **(C)** 5-year DCA curve. RFS, recurrence-free survival.
