# Supplementary figures and images for: Prognostic model for predicting recurrence in hepatocellular carcinoma patients with high systemic immune-inflammation index based on machine learning in a multicenter study
Source: Front Immunol. 2024 Sep 9;15:1459740. doi: 10.3389/fimmu.2024.1459740 (PMC11416987; doi:10.3389/fimmu.2024.1459740)

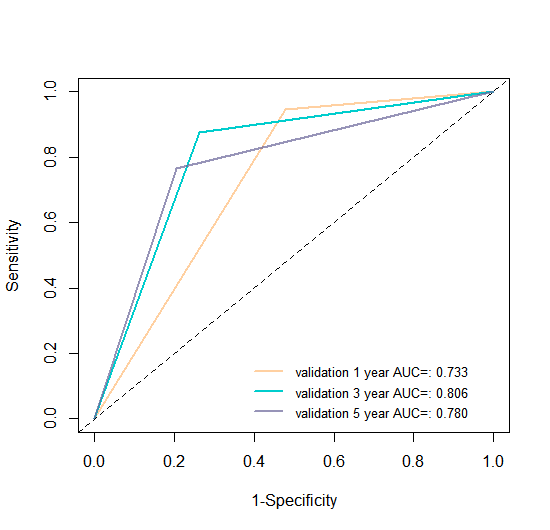

Supplement: Supplementary Figure 1 — Receiver operating characteristic (ROC) curves of the nomogram in the internal validation cohort. AUC, area under the curve. [file Image1.tiff]

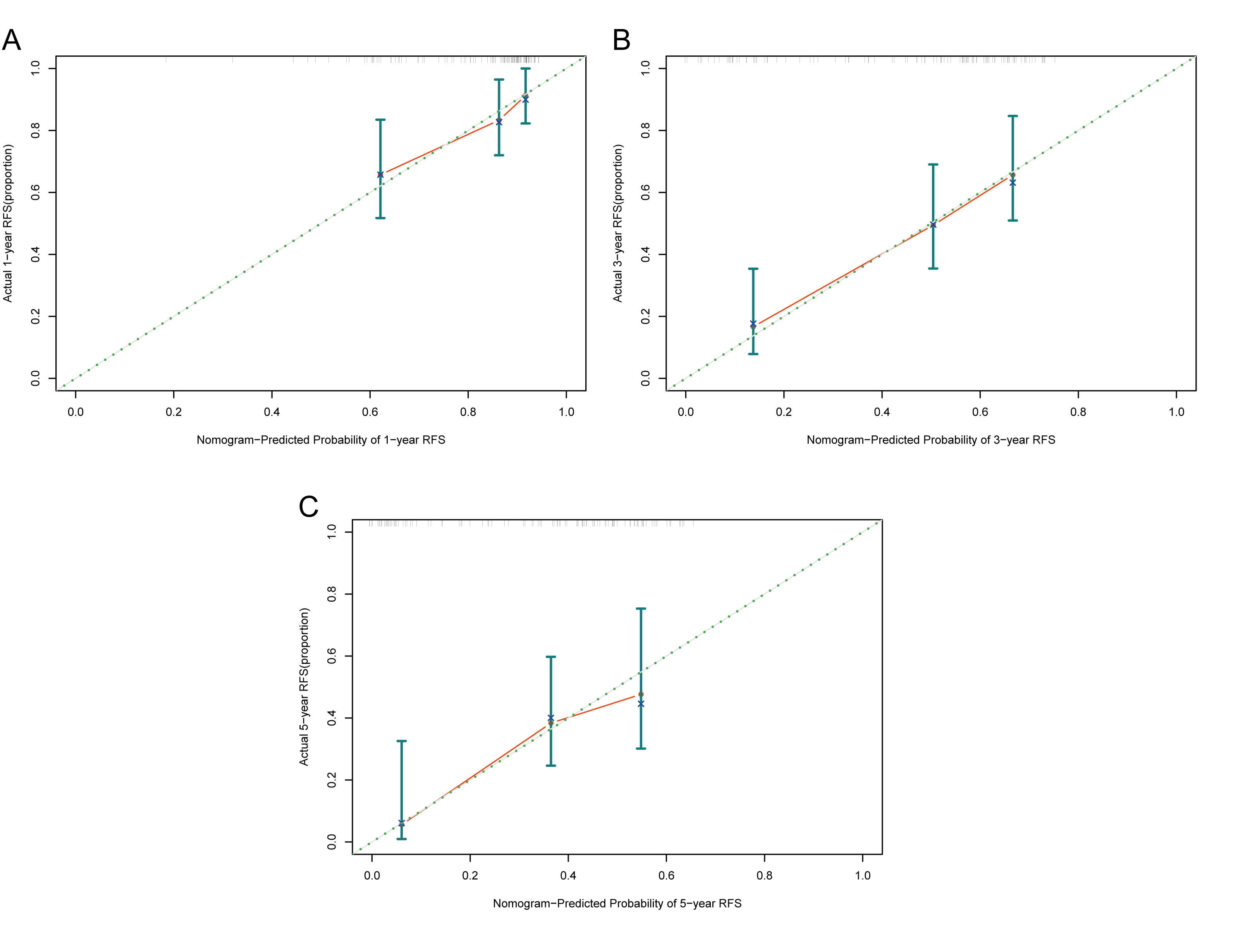

Supplement: Supplementary Figure 2 — Calibration curves of the nomogram in the internal validation cohort. (A) 1-year calibration curve. (B) 3-year calibration curve. (C) 5-year calibration curve. RFS, recurrence-free survival. [file Image2.tif]

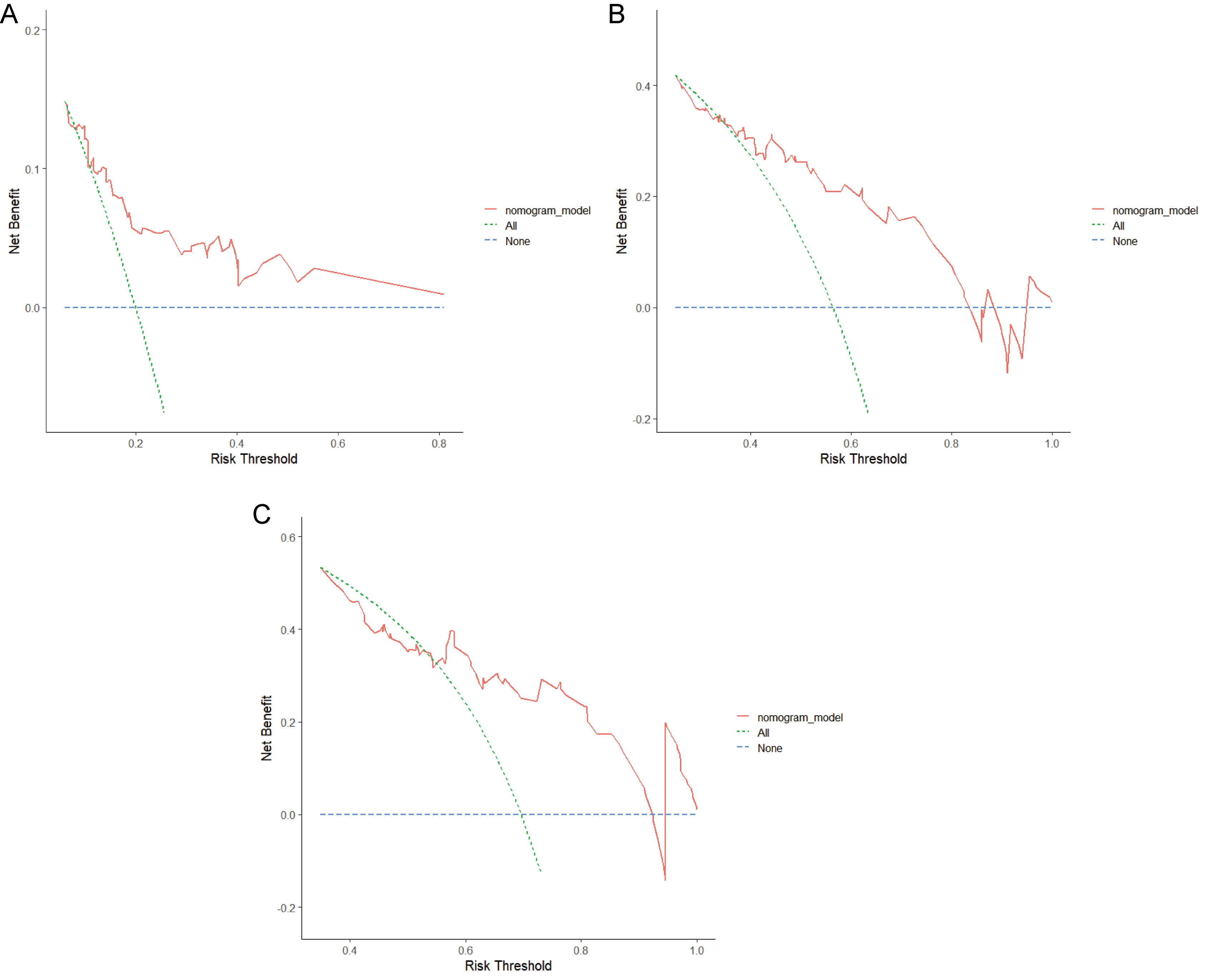

Supplement: Supplementary Figure 3 — Decision curve analysis (DCA) of the nomogram in the internal validation cohort. (A) 1-year DCA curve. (B) 3-year DCA curve. (C) 5-year DCA curve. RFS, recurrence-free survival. [file Image3.tif]

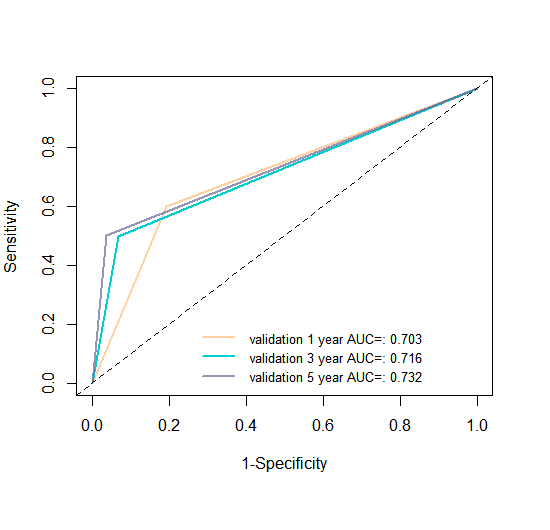

Supplement: Supplementary Figure 4 — Receiver operating characteristic (ROC) curves of the nomogram in the external validation cohort. AUC, area under the curve. [file Image4.tiff]

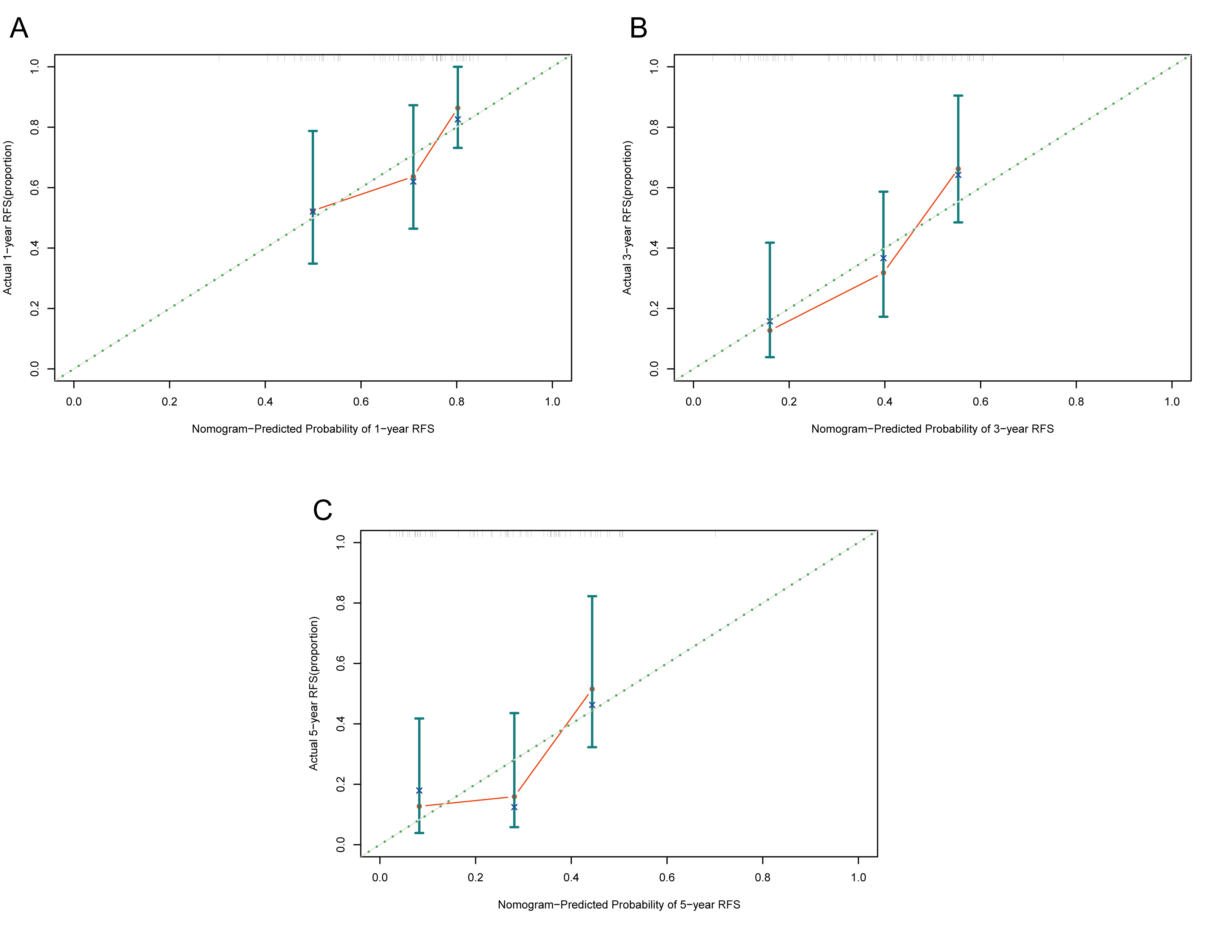

Supplement: Supplementary Figure 5 — Calibration curves of the nomogram in the external validation cohort. (A) 1-year calibration curve. (B) 3-year calibration curve. (C) 5-year calibration curve. RFS, recurrence-free survival. [file Image5.tif]

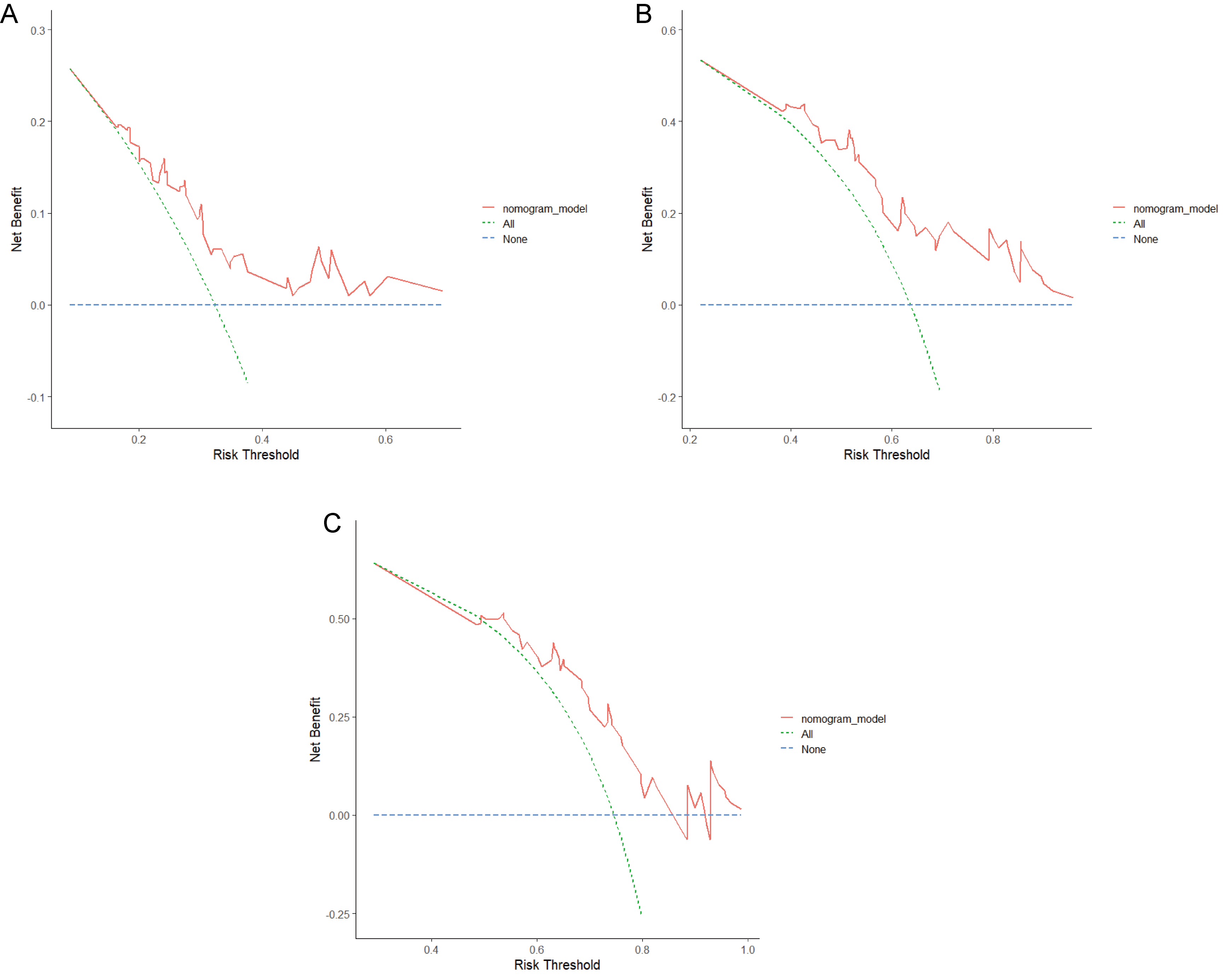

Supplement: Supplementary Figure 6 — Decision curve analysis (DCA) of the nomogram in the external validation cohort. (A) 1-year DCA curve. (B) 3-year DCA curve. (C) 5-year DCA curve. RFS, recurrence-free survival. [file Image6.tif]
